# Simulating ventricular systolic motion in a four-chamber heart model with spatially varying robin boundary conditions to model the effect of the pericardium

**DOI:** 10.1016/j.jbiomech.2020.109645

**Published:** 2020-03-05

**Authors:** Marina Strocchi, Matthias A.F. Gsell, Christoph M. Augustin, Orod Razeghi, Caroline H. Roney, Anton J. Prassl, Edward J. Vigmond, Jonathan M. Behar, Justin S. Gould, Christopher A. Rinaldi, Martin J. Bishop, Gernot Plank, Steven A. Niederer

**Affiliations:** a Department of Biomedical Engineering, School of Biomedical Engineering and Imaging Sciences, King’s College London, London, UK; b Department of Biophysics, Medical University of Graz, Graz, Austria; c University of Bordeaux, Talence, France; d LIRYC Electrophysiology and Heart Modeling Institute, Campus Xavier Arnozan, Pessac, France; e Cardiology Department, Guys and St Thomas’ NHS Foundation Trust, London, UK

**Keywords:** Computer models, Cardiac electromechanics, Ventricular systolic motion, Heart failure, Pericardium, Apico-basal shortening

## Abstract

The pericardium affects cardiac motion by limiting epicardial displacement normal to the surface. In computational studies, it is important for the model to replicate realistic motion, as this affects the physiological fidelity of the model. Previous computational studies showed that accounting for the effect of the pericardium allows for a more realistic motion simulation. In this study, we describe the mechanism through which the pericardium causes improved cardiac motion. We simulated electrical activation and contraction of the ventricles on a four-chamber heart in the presence and absence of the effect of the pericardium. We simulated the mechanical constraints imposed by the pericardium by applying normal Robin boundary conditions on the ventricular epicardium. We defined a regional scaling of normal springs stiffness based on image-derived motion from CT images. The presence of the pericardium reduced the error between simulated and image-derived end-systolic configurations from 12.8±4.1 mm to 5.7±2.5 mm. First, the pericardium prevents the ventricles from spherising during isovolumic contraction, reducing the outward motion of the free walls normal to the surface and the upwards motion of the apex. Second, by restricting the inward motion of the free and apical walls of the ventricles the pericardium increases atrioventricular plane displacement by four folds during ejection. Our results provide a mechanistic explanation of the importance of the pericardium in physiological simulations of electromechanical cardiac function.

## 1 Introduction

In the normal heart, as the electrical stimulus travels to the ventricular myocardium, ventricular myocytes depolarise and cellular contraction starts. Due to the highly organized helical structure of the myofibres across the ventricular walls, microscopic cellular shortening translates to a macroscopic shortening, thickening and twisting of the myocardium. When ventricles shorten, the atrioventricular plane undergoes a significant downward displacement (Rushmer et al., 1956), while the radial motion of the epicardial surface of the heart is restricted by the pericardium, a sack of fibrous tissue surrounding the heart, and its elastic anchoring to the surrounding tissues (Shabetai et al., 1979).

Computer models are increasingly used to study factors affecting cardiac electrical activation and mechanical contraction in healthy and diseased states. As simulating physiological systolic motion is fundamental for these models, boundary conditions imposed on the mechanics simulation are very important (Niederer et al., 2009, Peirlinck et al., 2019, Aguado-Sierra et al., 2011, Kayvanpour et al., 2015, Trayanova et al., 2011, Trayanova, 2011, Gurev et al., 2011). Previous three-dimensional computational models for cardiac electromechanics were mainly focused on the ventricles (Lee et al., 2018, Okada et al., 2017, Sermesant et al., 2012, Kerckhoffs et al., 2010, Tobon-Gomez et al., 2013). Although such models were extensively used to analyse ventricular systolic function (Crozier et al., 2016, Niederer et al., 2010), they have limitations in reproducing physiological systolic motion, as excluding atria and the major vessels from the geometry requires fixing the base of the ventricles. This contrasts with reality where the base moves significantly during ventricular contraction (Rushmer et al., 1956).

Recently, electromechanical models started moving from biventricular geometries to four-chamber geometries (Augustin et al., 2016b, Baillargeon et al., 2014, Fritz et al., 2014, Pfaller et al., 2018, Santiago et al., 2018, Krishnamurthy et al., 2016). One of the first high-resolution four-chamber geometry including the atria and a portion of the major vessels was presented by Augustin et al. (Augustin et al., 2016b). Free mechanical contraction was simulated by fixing the terminal ring of the major arteries and veins, while the motion of the apex was reduced by adding a cushion attached to the apical region. Although introducing the cushion at the apex provided an alternative to fixing the base and allowed simulations with a more physiological apico-basal shortening, the cushion tends to introduce artificial strains and is not justified by the presence of any anatomical structure, as in the heart the apex has no rigid attachment to the pericardium despite the pericardium being anchored to the diaphragm at the apical region. Furthermore, inward motion of the lateral walls was not constrained as the effect of the pericardial sack was not included in the simulation.

The majority of three-dimensional electromechanics models discards the effect of the pericardium due to the cost of implementing contact mechanics. Notably, Fritz et al. used contact mechanics to model the effect of the pericardium (Fritz et al., 2014). In this work, the tetrahedral mesh was extended to also include the surrounding tissue and the pericardium. In addition to fixing the terminal ring of the major vessels, they also fixed the apex, despite the absence of any physical link between the ventricle apex and the pericardium. The resulting motion was much more physiological due to the inclusion of the pericardium, however, the effect of fixing the apex was not studied. An alternative approach to solving the contact mechanics problem between the epicardium and the pericardium is to model the effect of the pericardium only, by limiting displacement of the epicardium normal to the surface (Chabiniok et al., 2012, Hirschvogel et al., 2017, Marchesseau et al., 2013, Moireau et al., 2012, Pfaller et al., 2018, Santiago et al., 2018, Tobon-Gomez et al., 2013, Ponnaluri et al., 2019). Regardless of the method used to simulate the effect of the pericardium, these studies showed that accounting for it in the simulation improves systolic motion. However, the physiological mechanisms through which this happens remain undetermined.

In this paper, we described the mechanism through which the pericardium causes improved cardiac motion, and studied the impact of the pericardium on local ventricular anatomy and regional strain. We ran simulations on a four-chamber geometry with and without the effect of the pericardium to observe the differences in systolic motion. We then compared the resulting and the image-derived displacement fields. We also performed a parametric study to investigate the effect of active tension parameters, boundary conditions and spring stiffness magnitude and scaling on both models with and without the effect of the pericardium.

## 2 Materials and methods

We briefly describe the construction of the four-chamber geometry and the main features of our electromechanics model, created using data acquired from a CRT patient with left bundle branch block. Details about the segmentation and the electromechanics model are provided in the supplement.

### 2.1 Four-chamber geometry

The four-chamber geometry was segmented from the end-diastolic (ED) CT image, as described previously by (Crozier et al., 2016). The resulting geometry was discretized with tetrahedra with a target edge length of 1 mm using CGAL (http://www.cgal.org/). A rule-based method was used to assign fibres in the ventricles with a fibre angle of 80° and -60° on the endocardial and the epicardial surface, respectively (Bayer et al., 2012, Land and Niederer, 2018). The sheet direction was set to -65° on the endocardium and 25° on the epicardium (Bayer et al., 2012). Atrial fibres were generated by mapping a pre-existing atrial fibre field onto our LA and RA endocardium and epicardium (Labarthe et al., 2014, Roney et al., 2018a, Roney et al., 2018b). Transmural fibre orientation was then interpolated linearly between these two surfaces.

### 2.2 Image-based kinematic reference model

**Fig. 1:**
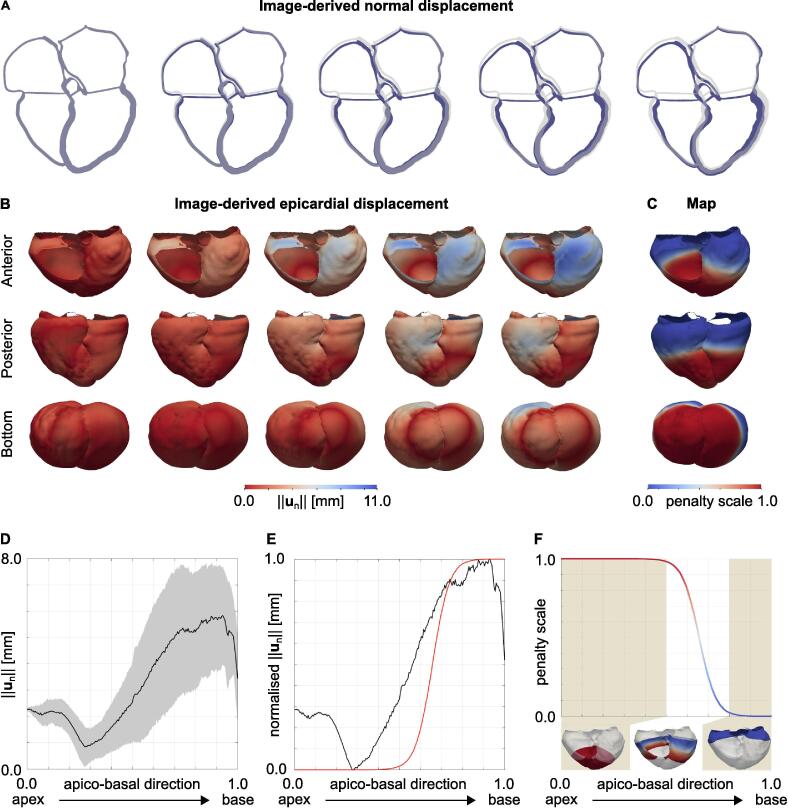
A Target systolic motion. The images represent the motion of a slice of the patient’s heart during systole as computed by the motion tracking algorithm (blue moving geometry). The grey geometry represents the ED geometry. B Epicardial displacement. The images show the distribution of the displacement of the epicardium normal to the surface during systole in an anterior (top row), posterior (middle row) and bottom (bottom row) view. C Penalty map. The figure shows the anterior, posterior and bottom view of the penalty map for the displacement normal to the surface applied on the epicardium of the ventricles to model the effect of the pericardium on the ventricles. D Apex to base epicardial displacement. The plot shows the epicardial displacement normal to the surface against the apico-basal direction (0 at the base and 1 at the apex). The black line shows the average trend, while the gray area shows the standard deviation. E Apex to base normalised epicardial displacement. The plot shows the epicardial displacement normal to the surface normalised between 0 and 1, together with the function we used to define the scale for the spring stiffness (red line). F Apex to base penalty map. The plot shows the penalty scale we derived from the data against the apico-basal direction. The function was computed by flipping the red curve shown in panel E. Epicardial regions with low and high displacement normal to the surface were applied with maximum and minimum penalty, respectively.

In order to visualise the patient’s heart motion, we applied a motion tracking algorithm to the reconstructed retrospective gated CT with ten frames. We assigned each voxel of each frame with a displacement with respect to ED (Shi et al., 2013). Then, the ED tetrahedral mesh was warped according to the resulting displacement field. This provided an image-driven deforming mesh that quantified the motion of the heart, which will also be the target motion for our simulations. Fig. 1, Fig. 1B show the image-derived patient’s heart motion during ventricular systole and the distribution of the surface normal displacements, respectively. The blue mesh represents the ED mesh deformed according to the image-derived displacement field, while the grey geometry represents the ED mesh. Fig. 1D shows the distribution in normal displacement in the apico-basal direction (0 at the apex and 1 at the base of the ventricles). The black line and the gray area represent the average displacement and the standard deviation, respectively.

### 2.3 Cardiac electromechanics model

The electrical activation of the ventricles was modelled with a reaction-eikonal model (Neic et al., 2017). Ventricular activation was initiated at the right ventricular (RV) endocardium. The conduction velocity of the ventricles was tuned to approximate the QRS duration of the patient. We excluded the atria and all the other labels from the eikonal solve to prevent activation.

Large deformations of the heart throughout the cardiac cycle were modelled with the finite elasticity equations solved in a Lagrangian reference frame (Nordsletten et al., 2011). The myocardium was modelled as a hyperelastic and nearly incompressible material. For ventricular passive mechanics, we used the transversely isotropic Guccione law and the orthotropic Usyk law (Guccione et al., 1991, Usyk et al., 2000). Passive mechanics of non-ventricular tissue was modelled with the neo-Hookean model to represent non-linear response of the material to external load. Tissue incompressibility was enforced with a penalty method (Flory, 1961, Ogden, 1978). Active contraction was modelled using a variant of a previous phenomenological contraction model (Niederer et al., 2010). Active contraction parameters were manually tuned to approximate the measured pressure–volume loop for both simulations in the presence and absence of the pericardium.

#### 2.3.1 Boundary conditions

**Fig. 2:**
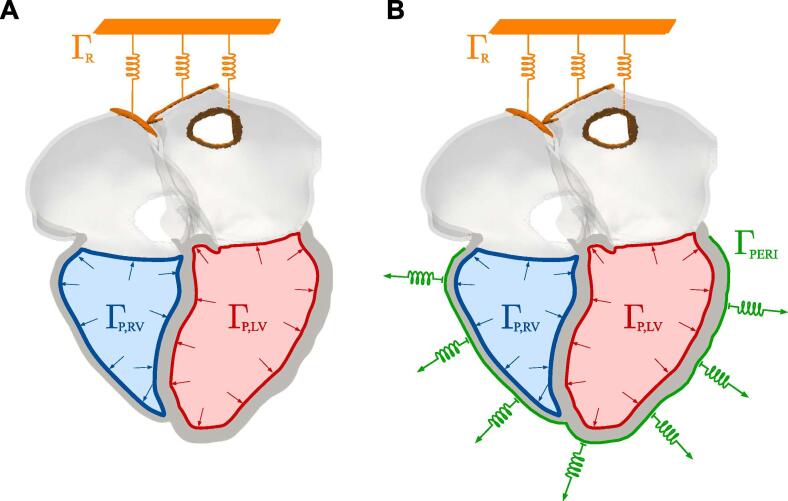
Boundary conditions. Boundary conditions applied in the simulation without A and with B the effect of the pericardium. We applied omni-directional springs at the two cropped right pulmonary veins and at the cropped superior vena cava, represented by the orange boundary Γ_R_. Neumann boundary conditions for left and right ventricular pressure were applied at the left ventricular endocardium Γ_P,LV_ (red) and at the right ventricular endocardium Γ_P,RV_ (blue), respectively. In the simulation with the pericardium, we added normal springs on the epicardial surface of the ventricles Γ_PERI_, shown in green. (For interpretation of the references to colour in this figure legend, the reader is referred to the web version of this article.)

Fig. 2 shows the boundary conditions we applied for simulations in the absence (A) and in the presence (B) of the pericardium. We constrained the simulation by applying omni-directional spring boundary conditions at the cropped right pulmonary veins and superior vena cava (orange boundaries), with a spring stiffness of 10.0 kPa mm^−1^ to reduce motion (Land and Niederer, 2018, Pfaller et al., 2018). The pressure–volume relationship in the ventricles was simulated as described previously (Augustin et al., 2016a). Ventricular afterload during ejection was represented with three-element Windkessel models (Westerhof et al., 2009). The parameters for left ventricular (LV) afterload were tuned to match the patient’s ejection fraction. For details about preload and afterload models, refer to Section 1.3.3 of the Supplement.

To simulate the effect of the pericardium, we applied normal springs to the epicardium of the ventricles (green boundary in Fig. 2B), using as a reference configuration the ED geometry (Pfaller et al., 2018). Fig. 1D shows the epicardial displacement plotted against the apico-basal direction. We observed that epicardial regions close to the base of the ventricles move normal to the surface. We therefore normalised the average displacement between 0 and 1 and transformed it into a function relating the epicardial displacement to the position along the apico-basal direction (red curve in Fig. 1E). We then flipped this function and turned it into a regional scaling of spring stiffness relative to the apico-basal direction (Fig. 1F). We applied a maximum spring stiffness of 50.0 kPa mm^−1^ and we scaled this value according to the map shown in Fig. 1C.

#### 2.3.2 Parametric study

We performed a parametric study to quantify the effect of active tension parameters on the model output. For both simulations with and without the effect of the pericardium, we ran simulations with: 20% increase in ventricular fibre active stress; 20% increase of duration of ventricular contraction; 40% of fibre active stress applied in the transverse plane (Walker et al., 2005, Genet et al., 2014); 20% increase in ventricular fibre active stress and 40% of fibre active stress applied in the transverse plane. We computed the percentage change from the control simulations of the outputs shown in Table 1.

**Table 1:** **Parameteric study.** The table shows the simulation output for different parameter settings. The control simulations both with and without the pericardium are shown in the gray rows. Left ventricle (LV); right ventricle (RV); ejection fraction (EF); peak pressure (PP), atrioventricular plane displacement (AVPD) (positive means downwards); ventricular apex displacement (VAD) (positive means upwards); isovolumic contraction (IVC); ejection (Ej); fibre active stress (T_f_); duration of contraction ($\tau_{\mathrm{dur}}$); transverse active stress (T_sn_); aortic resistance (Z); end-diastolic pressure (EDP); aortic pressure (P_Ao_); veins omni-directional spring stiffness (k) in kPa mm^−1^; pericardium normal spring stiffness (k_n_) in kPa mm^−1^.

We used the same model outputs to study the effect of the boundary conditions on both models with and without the pericardium. We ran simulations with: 20% increase of aortic and pulmonary artery resistance; 20% increase of LV and RV end-diastolic pressure; 20% increase in aortic and pulmonary artery pressure at ejection; omni-directional springs applied to all cropped veins. For the simulation with the effect of the pericardium, we also decreased by ten fold the stiffness applied at all cropped veins. We used the model with the effect of the pericardium to study the effect of the normal spring stiffness, applied at ventricular epicardium. We decreased the stiffness of the normal springs by two, ten and fifty fold. To investigate the effect of the spring stiffness regional scaling, we ran simulations with uniform spring stiffness on the whole ventricular epicardial surface with normal spring stiffness set to the control value (50.0 kPa mm^−1^) and fifty fold lower.

## 3 Results

We first compare simulation results in the absence and presence of the pericardium on the four-chamber heart. We then show the comparison of the simulated and the image-derived end-systolic configuration. We investigate the effect of active tension parameters and boundary conditions on the model.

### 3.1 Effect of the pericardium on systolic motion

**Fig. 3:**
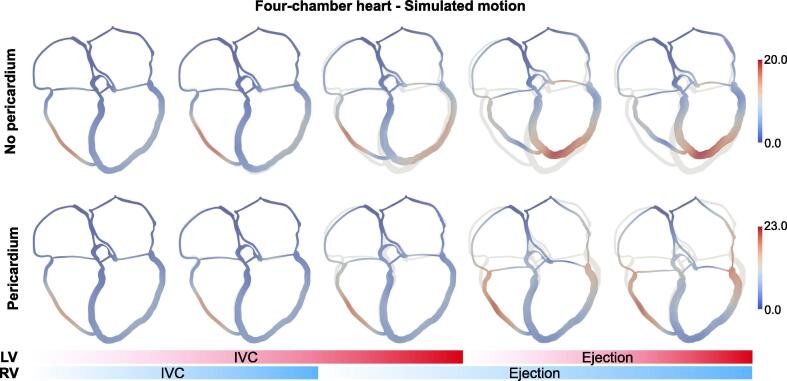
Simulated motion. The images show the motion of a slice of the four-chamber geometry for the simulation without (top row) and with (bottom row) the pericardium. The grey geometry represents the ED configuration, while the moving geometry is coloured according to the displacement magnitude. The blue and red bars at the bottom display the phase of the cardiac cycle for the RV and the LV, respectively. (For interpretation of the references to colour in this figure legend, the reader is referred to the web version of this article.)

Fig. 3 shows the motion of a slice of the four-chamber heart without (top row) and with (bottom row) the effect of the pericardium. In the absence of the pericardium, the ventricles deform to a more spherical shape during IVC (third frame). As ventricular contraction starts and cavity pressure increases, the lateral walls move outwards and the apex moves upwards as the cavity volume has to remain constant. As ejection starts, the apex keeps on moving upwards and the lateral walls move inwards. In the presence of the pericardium, the heart does not spherise during IVC as the pericardium prevents the lateral walls from bulging out, and therefore we do not observe apico-basal shortening of the geometry. During ejection, the lateral walls slide on the pericardium with little inward motion and the base moves downwards, while the apex remains mainly static. The pericardium also affects septal motion. In the absence of the pericardium, the septum bulges towards the RV. In the presence of the pericardium the septum is pushed towards the LV at early systole and then pushed back towards the RV at late systole. By visual comparison with systolic image-derived motion shown in Fig. 1A, we can conclude that including the effect of the pericardium allows us to obtain a motion that is qualitatively more similar to the patient’s motion. In addition, we investigated the effect of the pericardium on local LV endocardial curvature, LV wall thickening and strains. We showed that strains and wall thickening were insensitive to the presence of the pericardium. On the other hand, local curvature changed significantly. We described detailed results in Section 2.2 of the Supplement.

### 3.2 Comparison with image-derived end-systolic displacement

**Fig. 4:**
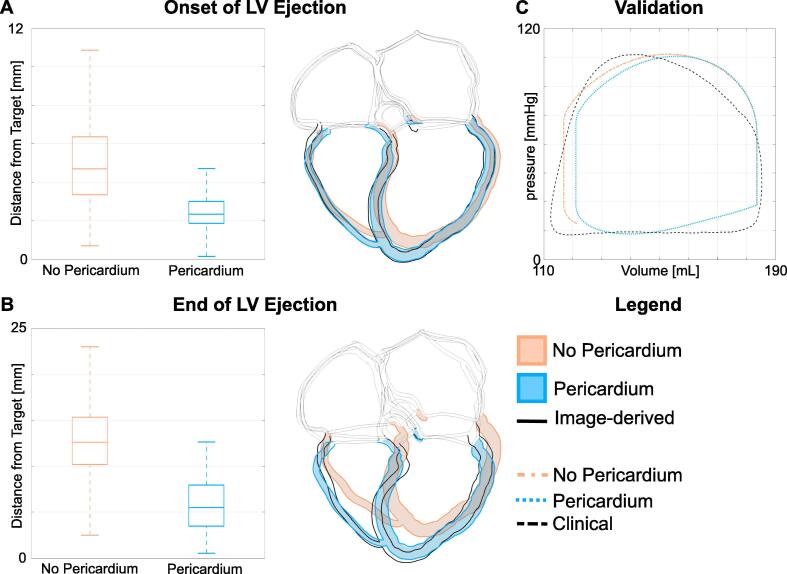
A, B Comparison with image-derived displacement. We show the comparison between the image-derived and the simulated motion. Figure A and B show the comparison at the onset of LV ejection and at the end of LV ventricular systole. For each figure, On the left we show the boxplots of the Euclidean distance between the image-derived and the simulated motion without (orange) and with (light-blue) the effect of the pericardium. On the right, we overlapped the image-derived configuration (geometry with black borders) to the configurations simulated without (orange) and with (light-blue) the effect of the pericardium. C Pressure-volue loop validation. The clinical pressure–volume loop (black dashed line) is compared to the simulated pressure–volume loops without (orange) and with (light-blue) the effect of the pericardium. (For interpretation of the references to colour in this figure legend, the reader is referred to the web version of this article.)

To quantify the impact and importance of the pericardium on the global and local deformations, we compared the configuration estimated by the image-derived motion to the simulated configurations in the presence and in the absence of the pericardium at the end of LV IVC and ejection. Fig. 4 shows the boxplots of the node-wise Euclidean distance of ventricular nodes between the mesh deformed according to the image-derived displacement field and the simulated deformed configuration in the presence and in the absence of the pericardium. We show comparison for the motion at the onset of contraction (Fig. 4)A and at the end of LV ejection (Fig. 4B). We also show a slice of the four-chamber heart at the corresponding phases of the cardiac cycle. Orange and light-blue boxplots and geometries represent results for the simulation in the absence and in the presence of the pericardium, respectively. Fig. 2 in the supplement shows a comparison between the image-derived end-systolic displacement and simulated end-systolic displacement in the presence and in the absence of the pericardium.

Including the effect of the pericardium reduces the error from the image-derived motion. In the absence of the pericardium (orange boxplots in Fig. 4A abd B) the mean distance from the image-derived configuration was 5.0±2.1 mm and 12.8±4.1 mm at the onset and at the end of LV contraction, respectively. On the other hand, in the presence of the pericardium (light-blue boxplots in Fig. 4A and B) the mean error from the image-derived motion decreased to 2.9±2.0 mm and 5.7±2.5 mm at the onset and at the end of LV ejection, respectively. The comparison between clinical measurements and the simulated pressure–volume loop of Fig. 4C show that simulated pressure and volume with (light blue) and without (orange) the effect of the pericardium fall within physiological ranges.

### 3.3 The effect of active contraction parameters and boundary conditions on simulated dynamics

Table 1 shows the effect of active tension parameters (sections labelled as T act) and boundary conditions (sections labelled as BCs) on model outputs. The rows represent different simulations and the columns represent different output metrics. The first column shows the parameter that was altered for each simulation. Each cell contains the simulation output computed for each simulation. The percentage change from the control simulation are reported in brackets. Control simulations both without and with the effect of the pericardium are highlighted in the gray rows.

Active stress parameters and boundary conditions have similar effects on EF and PP in the presence and in the absence of the pericardium. In the absence of the pericardium, increased EF leads to larger apical motion. On the other hand, in the presence of the pericardium apical motion remains small and AVPD increases. Introducing transverse stress decreases ventricular EF in both models. Although increasing the fibre active stress by 20% led to similar EF to the control simulations, systolic apical motion remained large without the pericardium. We were therefore able to conclude that our observations about apical motion were not dependent on active tension parameters and boundary conditions other than the pericardium.

We performed a study of the effect of spring stiffness and regional scaling on simulated motion in the presence of the pericardium. Reducing spring stiffness with regional scaling leads to increased unphysiological apical motion. In particular, apical motion during IVC increases almost by three folds with the smallest spring stiffness used, as the lateral walls of the ventricles are allowed to move outwards and therefore the ventricles spherise. Applying a uniform normal spring stiffness significantly decreases ejection. To increase ventricular EF, we reduced spring stiffness by 50-fold. Although this increased EF, systolic apical motion increased as a consequence. This parametric study allowed us to observe that a regional scaling of spring stiffness representing regional forces exerted by the pericardium on ventricular epicardium are required to reduce the constraint on the base, allowing for significant AVPD and small apical motion.

## 4 Discussion

We used a four-chamber electromechanics model to study the effect of the pericardium on cardiac motion. As shown previously, including the effect of the pericardium improved simulated systolic motion, bringing it closer to the image-derived motion. The pericardium prevents the heart from spherising during IVC and prevents the apex from moving upwards during ventricular ejection. We showed that our conclusions are independent from active contraction magnitude and duration, and end-diastolic and aortic boundary conditions. We also observed significant differences in septal motion and local end-systolic curvature change. On the other hand, local wall thickening and strain distribution were insensitive to the presence of the pericardium, leading us to the conclusion that strain distribution might be a more robust measurement for model validation in models that do not include a pericardium constraint.

The addition of the pericardium improved model predictions of septal and apical motion, which are potential indicators of patient response to cardiac resynchronization therapy (CRT) and should be recapitulated by the models (Abbasi et al., 1974, Ghani et al., 2015, Stankovic et al., 2015). The pericardium leads to simulations with physiological systolic motion, with the apex remaining mainly static. Our results agree with observation on patients with congenital (Connolly et al., 1995, Payvandi and Kerber, 1976, Wilson et al., 2014) or acquired absence (Payvandi and Kerber, 1976) of the pericardium, experiencing cardiac hyper-mobility, swinging motion and large upwards motion of the apex (Connolly et al., 1995, Psychidis-Papakyritsis et al., 2007). (Holt et al., 1960) observed an increase in pressure in the pericardial space at the ventricular midwall during IVC. They attributed this effect to the ventricles trying to deform to a more spherical shape, as we observed in our simulations. Due to its elliptical shape, the cavity volume has an approximately quadratic dependence on the radius and a linear dependence on the apex-basal distance. This amplifies the effects of radial wall motion on the apex in the absence of the pericardium, with an average displacement normal to the surface of only 2.3 mm of the LV free wall causing an apical displacement of 10.5 mm, as shown in the third frame in top panel of Fig. 3. Holt et al. also measured negative pericardial pressure during ventricular ejection (Holt, 1970). The pericardium therefore constrains the inwards motion of the epicardial surface due to a suction effect. Ventricles were also reported to spherise during IVC in a computational study by (Nickerson et al., 2005), simulating LV mechanical contraction on a LV geometry in the absence of the pericardium. Our results show that this occurs even if the atria and major vessels are included in the mesh, and the base is not rigidly constrained. Abnormal septal motion anteriorly towards the RV free wall was observed in the absence of the pericardium, consistent with our simulation (Connolly et al., 1995, Payvandi and Kerber, 1976). In the presence of the pericardium, the simulated septal motion is consistent with septal motion reported in patients with dyssynchronous activation (Bevans and Rapaport, 1973).

Previous computational studies investigating the effect of the pericardium reported greatly improved cardiac motion in the presence of the pericardium in four-chamber heart (Fritz et al., 2014, Pfaller et al., 2018) and LV models (Ponnaluri et al., 2019). (Fritz et al., 2014) simulated the pericardium as a contact mechanics problem, which significantly increases the computational costs. Other studies used a simplified approach with normal springs, similarly to this study (Pfaller et al., 2018, Ponnaluri et al., 2019). Pfaller et al. however used a uniform spring stiffness and a dashpot, but they included adipose tissue in the mesh. Spring boundary conditions were therefore applied on the convex hull and not directly on the epicardial surface of the ventricles close to the base (Pfaller et al., 2018). We did not include adipose tissue in our mesh, but we derived a regional scaling for spring stiffness representing the local mechanical constraints exerted by the pericardium on the ventricles using clinical data. Despite the differences in modelling approaches to simulate the pericardium, our conclusions are in agreement with those reported by these studies.

### 4.1 Limitations

Our four-chamber simulations offer a valuable tool for studying the effect of the pericaridum on the ventricles, but they have some limitations. First, we did not use a close loop model. Although this may affect diastolic function, the model was not dependent on a simple sensitivity test of the applied diastolic boundary conditions. Secondly, we did not model the effect of the pericardium on the atria, even though the pericardium surrounds all the four chambers. We also simplified passive material properties of atrial myocardium as we ignored the effect of the fibres. These factors might have an effect on ventricular motion. However, the focus of our study is contraction in the ventricles, while atrial dynamics will predominately affect diastolic ventricular function.

We simplified the function defining how spring stiffness varies from apex to base. Due to this, we simulated a reduced inward motion of the epicardium of the ventricles while we overestimated the downwards motion of the base to achieve a more physiological ejection. Finally, our model for ventricular active tension generation does not account for length-dependence, although this might show additional differences between the simulations with and without the effect of the pericardium especially on local strains.

## 5 Conclusions

Our work provides a physiological explanation of the mechanisms by which the pericardium improves physiological systolic motion. We extended the work of (Augustin et al., 2016b) by adding pressure–volume dynamics in the ventricles to study the effect of the pericardium on the ventricles. Our simulations show that including the pericardium in the simulation reduces the error between simulated and image-derived motion. The pericardium hinders the outward motion of the free walls during IVC. The iso-volumetric constraint of the LV cavity then prevents the apex from moving upwards. During ejection, the pericardium reduces inward motion of the LV walls, limiting circumferential contraction. As the myocytes are preferentially aligned in the longitudinal direction, cellular contraction leads to a macroscopic apico-basal shortening, which results in myocardium thickening due to incompressibility of cardiac tissue. Although our results do not show significant effects of the pericardium on local strain distribution and wall thickening, we showed that the pericardium affects systolic motion and consequently end-systolic curvature change. This might in turn affect simulated LV function and therefore the predictive power of the model.

## Declaration of Competing Interest

The authors have no conflict of interests.
