# Supplementary material for: Simulating ventricular systolic motion in a four-chamber heart model with spatially varying robin boundary conditions to model the effect of the pericardium
Source: J Biomech. 2020 Mar 5;101:109645. doi: 10.1016/j.jbiomech.2020.109645 (PMC7677892; doi:10.1016/j.jbiomech.2020.109645)
Supplement: Supplementary data 1 [file mmc1.pdf]

Supplement

The supplement is divided into two sections. Section 1 provides details about the electromechanics model on the four-chamber geometry. Section 2 shows additional results comparing simulations of the four-chamber heart with and without the effect of the pericardium.

1 Four-chamber heart electromechanics model

First, we described our pipeline to construct a four- chamber geometry from CT images. Then, the main features of our four-chamber electromechanics model are presented. All the electromechanics simulations described in the following sections were run with the Cardiac Arrhythmia Research Package (CARP) (Vigmond et al., 2008, 2003).

1.1 Patient data

The simulations were based on data from a 78-year-old female HF patient with a prolonged QRS duration of 172.8ms, derived from the patient's endocardial activation map. The data were previously presented in a cardiac resynchronization therapy (CRT) study (clinical trial registration No.: NCT01464502) (Behar et al., 2017). We specifically chose to use data from a CRT patient as this gave us access to retrospective gated CT reconstructed over ten frames over an entire cardiac cycle and invasive pressure measurements, that can be used to constrain the model. We computed the LV volume transient from the ten-frames CT. We interpolated the obtained data over time and we synchronised it to the catheter pressure measurements to obtain the patient's pressure-volume relationship.

1.2 Segmentation

End-diastolic (ED) CT images with in and out-plane resolution of 0.36mm and 0.5mm, respectively, were segmented to obtain a four-chamber geometry. First, blood pools for the ventricles, the atria and the aorta, LV myocardium and papillary muscles were segmented with an automatic segmentation tool (Zheng et al., 2008). The resulting segmentation was then post-processed using the open source software Seg3D (CIBC, 2016). The automatic segmentation tool failed to precisely segment the LV myocardium, LV papillary muscles and blood pool due to the presence of artefacts and poor contrast in the septum.

We thresholded the image intensity to generate labels representing the LV blood pool and the LV papillary muscles. To obtain the LV myocardium, we combined the labels for the LV blood pool and papillary muscles and dilated them by 6mm so that our segmentation matched the epicardium of the LV free wall. The right ventricular (RV) blood pool was divided into two parts to add a label for a portion of the pulmonary artery blood pool as well. The remaining RV blood pool was dilated by 3.5mm, to represent the RV myocardium in line with values for RV wall thickness reported in the literature (Ho and Nihoyannopoulos, 2006; Matsukubo et al., 1977). The same procedure was applied to the blood pool of the left atrium (LA), the right atrium (RA), the aorta and the pulmonary artery to obtain atrial myocardium, aortic and pulmonary arterial walls with a wall thickness of 2mm (Beinart et al., 2011; Mensel et al., 2013). The pulmonary veins, the LA appendage (LAA), the superior vena cava and the inferior vena cava were cut perpendicular to the flow direction. Labels were added at the cropped veins to apply boundary conditions. Endocardial surfaces of ventricles and atria were closed by adding 2mm-thick valve planes at each inlet and outlet of the chambers (Fritz et al., 2014; Land and Niederer, 2018). The resulting segmentation was upsampled with an isotropic resolution of 150µm and smoothed with a variational approach (Knoll et al., 2012; Crozier et al., 2015).

1.3 Cardiac electromechanics model

1.3.1 Electrical activation

The electrical activation of the heart was modelled with a reaction-eikonal model (Neic et al., 2017). Ventricular myocardium was modelled as a transversely isotropic conduction medium with a conduction velocity (CV) in the fibre and in the transverse direction of $0.67 \mathrm{m\,s^{-1}}$ and $0.27 \mathrm{m\,s^{-1}}$, respectively, set to match the QRS duration of the patient. An isotropic, one-element thick fast endocardial conduction (FEC) layer was added in the endocardial surface of the LV with CV two times higher than the bulk CV of the ventricles to simulate fast endocardial activation (Hyde et al., 2015; Lee et al., 2019). We excluded the atria and all the other labels from the eikonal solve to prevent activation. The endocardial surface of the RV was stimulated to initiate ventricular activation. The RV stimulus was located on the endocardium of the RV free wall based on the lead location in the patient's heart, determined from the CT images. To simulate LBBB activation typical of patients with a broad QRS and dyssynchrony, the LV was not stimulated (Strik et al., 2012).

1.3.2 Mechanical contraction

Large deformations of the heart throughout the cardiac cycle were modelled with the finite elasticity equations solved in a Lagrangian reference frame (Nordsletten et al., 2011). The myocardium was modelled as a hyperelastic and nearly incompressible material. For ventricular passive mechanics we used a transversely isotropic Guccione constitutive law (Guccione et al., 1991):

$$
\psi(\mathbf{E}) = \frac{C}{2}\left(e^{Q}-1\right) + \frac{k}{2}\ln^2(J) \ ,
$$
$$
Q = b_{ff}E_{ff}^2 + 2b_{fs}(E_{fs}^2 + E_{fn}^2) + b_{ss}(E_{ss}^2 + E_{nn}^2 + 2E_{sn}^2) \ ,
$$

where ψ is the strain energy function and C, b_f, b_{fs} and b_{ss} are the material parameters. The f, s and n in the Cauchy-Green strain tensor $\mathbf{E}$ represent the strain in the fibres, sheet and normal to sheet directions, while J indicates the Jacobian determinant of the deformation gradient tensor. Parameters for ventricular stiffness were set based on parameters estimated in HF patients (Nasopoulou et al., 2017), see Table 1. Since transversely isotropic material laws do not reflect the underlying myocardium microstructure and may not provide a true representation of the myocardium stress-strain relations (Usyk et al., 2000), we investigated whether the difference on strain distribution observed between the simulations with and without the effect of the pericardium were dependent on the assumption of transverse isotropy. We therefore ran additional simulations with the Usyk material law, an extended version of the Guccione law to account for orthotropic material behaviour (Usyk et al., 2000):

$$
\psi(\mathbf{E}) = \frac{C}{2}\left(e^{Q}-1\right) + \frac{k}{2}\ln^2(J) \ ,
$$
$$
Q = b_{ff}E_{ff}^2 + b_{ss}E_{ss}^2 + b_{nn}E_{nn}^2 + 2(b_{fs}E_{fs}^2 + b_{fsn}E_{fn}^2) + b_{sn}E_{sn}^2) \ .
$$

We used the same values for C and b_{ff} of the Guccione law. We scaled the value for b_{ff} using the ratios computed from the data in the literature (Schmid et al., 2008). Table 1 shows the resulting values. Passive mechanics of non-ventricular tissue was modelled with a neo-Hookean model to represent non-linear response of the material to external load:

$$
\psi(\mathbf{E}) = c(I_1 - 3) + \frac{k}{2}\ln^2(J) \ ,
$$

where c is the only material parameter and I_1 denotes the first invariant of the Cauchy-Green strain tensor. In these tissues, we ignored the effect of the fibres. For atria, we used the average of the parameter values of the anterior and posterior regions reported in the literature (Di Martino et al., 2011), resulting in c = 7.45kPa. For the aorta and the pulmonary artery, we imposed c = 26.66kPa and c = 3.7kPa, the average parameter value of the two cases reported in previous works, respectively (Horný et al., 2006; Tian et al., 2016). Rings at the cropped pulmonary veins, superior vena cava, inferior vena cava and LAA were assigned with the same parameter value used for the atria. To restrict their deformation, the valve planes were assigned with a parameter c = 1000.0kPa. Tissue incompressibility was enforced with a penalty method (Flory, 1961; Ogden, 1978), with a penalty coefficient k = 1MPa. The parameters for passive mechanics of the four-chamber heart are summarized in the first section of Table 1.

Table 1: Material parameters used for passive mechanics in the four-chamber heart.

Geometry	Passive Material Parameters							
**Four-chamber heart:**								
	C	b_{ff}	b_{fs}	b_{fn}	b_{ss}	b_{sn}	b_{nn}	k
	kPa	-	-	-	-	-	-	MPa
Ventricles - Transversely isotropic	3.0	19.25	8.75		7.0			1.00
Ventricles - Orthotropic	3.0	19.25	5.7	5.41	7.96	4.37	4.74	1.00
	c							k
	kPa							MPa
Atria	7.45							1.00
Aorta	26.66							1.00
Pulmonary artery	3.7							1.00
Valve planes	1000.0							1.00
Cropped veins & LAA	7.45							1.00

Active tension in the ventricular myocardium was simulated with a phenomenological model (Kerckhoffs et al., 2003; Niederer et al., 2010). The activation time computed by the reaction-eikonal model was used to trigger the active tension transient in the ventricular myocardium. Since cardiac active contraction occurs predominantly in the fibre direction, the active stress computed by the cellular model was added to the passive stress in the fibre direction (Nordsletten et al., 2011). The duration of contraction was set to 550.0ms to match the systolic duration of the patient. The rising and the decay time were set to 130.0ms and 100.0ms, respectively, and we did not account for the effect of length-dependence. The peak in active tension for simulations with and without the effect of the pericardium was set to approximate the patient's pressure catheter measurements. In the model without the pericardium, we set the peak in active tension to 115.0kPa. In the model with the effect of the pericardium, ventricular shortening acts against atrial stiffness, as atria have to deform significantly to allow ventricular contraction. We increased the peak in active tension to 125.0kPa to match the peak in pressure of the patient.

1.3.3 Boundary conditions

The stress-free configuration was retrieved with a backward displacement method (Bols et al., 2013). We unloaded the ED geometry from an LV ED pressure of 18.0mmHg obtained from catheter measurements of the patient and a RV ED pressure of 9.54mmHg, obtained by scaling the LV ED pressure based on the ratio reported in the literature (Hyde et al., 2016). The unloaded configuration was inflated back to the ED geometry as a pre-step before starting the simulation of the cardiac cycle at ED. The pressure-volume relationship in the ventricles was simulated as described previously (Augustin et al., 2016). Figure 1A shows a schematic of the method we used, with q representing the flow. The unloaded configuration was inflated to the ED ventricular pressure as a prestep, and the simulation was started at ventricular ED. During IVC, the volume was kept constant by

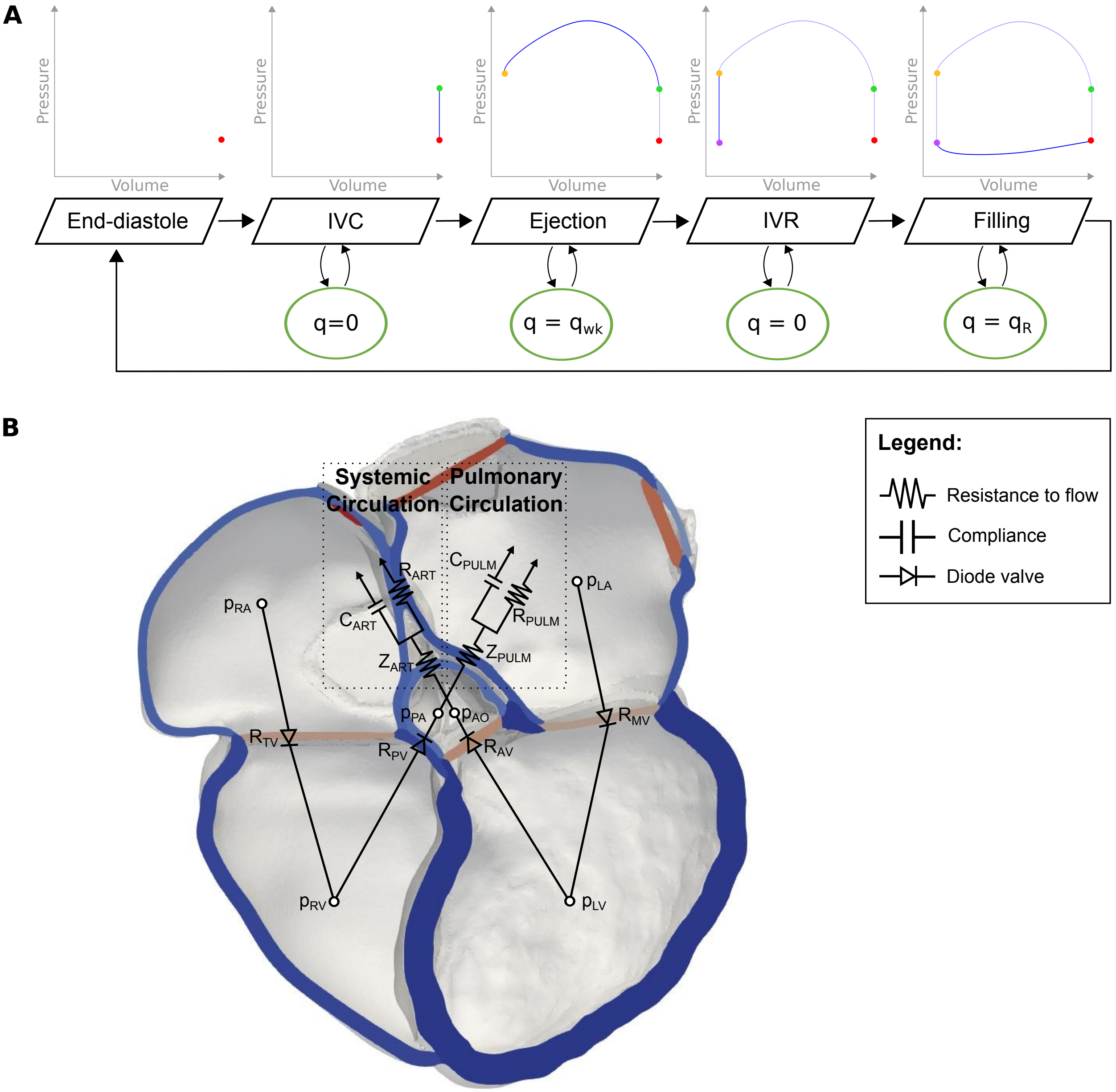

Figure 1: **A Coupling scheme.** The LV and RV pressures in the three-dimensional model were adapted to maintain a zero flow (q) during IVC and IVR, to match the Windkessel flow q_{WK} during ejection and transvalvular flow q_{R} during filling. The filling stops when the ED pressure is reached again. **B Preload and afterload model - Four-chamber heart.** The preload of the ventricles was modelled with constant LA and RA pressures. Ventricular afterload was modelled with two three-element Windkessel models, representing the systemic and the pulmonary circulations. The four cardiac valves were modelled as diodes.

recasting the formulation as a saddle point problem (Gurev et al., 2015). Once the LV pressure overcame the aortic pressure, initialised at 70.0mmHg (Blair et al., 1984), ejection started. RV ejection was initiated when the RV pressure overcomes the pulmonary arterial pressure, initialised at 15.0mmHg (Zapol and Snider, 1977) .

During ejection, the three-dimensional model was coupled with two three-element Windkessel models (Westerhof et al., 2009). Left ventricular afterload parameters were tuned prior the mechanics simulation. We solved the ODE for a three-element Windkessel model using the invasively measured pressure transient as an input. We then compared the resulting volume transient and the volume transient derived from the CT. We finally chose the set of parameters Z, R and C that provided the best fit using a statistical latin hypercube sampling. This resulted in the following parameters values: Z = 0.13mmHg s mL^{-1}, C = 0.85mL mmHg^{-1} and R = 5.76mmHg s mL^{-1}. The parameters in the active contraction model scale directly with organ scale phenotypes. This allows the reference tension to be scaled to match the desired peak pressure and the duration of contraction to be scaled to match the duration of ventricular systole. The reference tension and the duration of contraction were then scaled to match the patient's LV peak in pressure and LV systole duration. Since pressure data for the right ventricle were not available, we scaled the values for the left ventricles using scaling factors provided in the literature (Niederer et al., 2010). This lead to the following values for RV afterload parameters: Z = 0.05mmHg s mL^{-1}, C = 0.11mL mmHg^{-1} and R = 25.92mmHg s mL^{-1}. The aortic and the pulmonary valve were modelled as diodes with no forward resistance and a backward resistance of 1000.0mmHg s mL^{-1} to prevent backflow.

Isovolumic relaxation (IVR) was treated similarly to IVC (Gurev et al., 2015). Finally, the filling phase was regulated by the mitral and the tricuspid valves, modelled as diodes with a forward and a backward resistance of 0.05mmHg s mL^{-1} and 1000.0mmHg s mL^{-1}, respectively. In our simulations, the solution of the atria and ventricle mechanics were solved in a single monolithic solver and so their mechanical interactions are captured. However, atria were passive with no endocardial pressure boundary conditions. Atrial dynamics play an important role in ventricular filling, while they have limited effects on ventricular systole as during this phase ventricular pressure is much higher than atrial pressure. We therefore decided to exclude atrial dynamics from the model to reduce model complexity. Any atrial dynamics would likely manifest as a change in the diastolic pressure. However, varying the end-diastolic pressure did not affect the study findings consistent with the atria not playing a significant role in the relationship between the pericardium and ventricular motion. Figure 1B shows the scheme for the preload and the afterload of the four-chamber heart. Since the simulation without the effect of the pericardium was more computationally expensive, we simulated only up to the end of LV IVR.

2 Additional results

First we show additional result for the effect of the pericardium on ventricular systolic displacement. Then, we show additional analysis of changes in systolic curvature, wall thickening and strain distribution due to the effect of the pericardium.

2.1 The effect of the pericardium on systolic motion

To quantify the impact and importance of the pericardium on the global and local deformations, displacements from both simulations were compared with image-derived displacements of the ventricles. We computed the displacement with respect to the ED geometry for the end-systolic geometry estimated by the motion tracking algorithm. We then normalised the displacement magnitude between 0 and 1. We did the same for the end-systolic deformed geometries simulated in the presence and in the absence of the pericardium. Figure 2A shows the normalised displacement magnitude of the models plotted versus the normalised image-derived displacement magnitude. If the point lays on the black dashed line, it means that model prediciton matches the image-derived displacement. Light and dark purple points show results for the simulation with and without the effect of the pericardium, respectively. The data points were enclosed by convex hulls, with squares and circles representing the simulation without and with the pericardium, respectively. Figure 2B shows the same results divided in different regions: the apex, the base of the LV and the base of the RV. Figure 2C shows a slice of the four-chamber geometry, where the highlighted regions are colour-coded according to the Figure 2B.

As discussed in the manuscript, The addition of the effect of the pericardium brings the motion closer to the image-derived motion. In the absence of the pericardium (dark purple) the nodes that should have a small displacement are predicted to move the most. On the other hand, in the simulation with the pericardium (light purple) ventricular nodes move in the right direction, with high displacement assigned to the nodes that move the most in reality. In the simulation without the pericardium the apex (dark green) is predicted to have high displacement, while it is largely static in the image-derived motion. In the simulation with the pericardium, the apical motion (light green) is significantly reduced. The simulation without the pericardium also underestimates the motion of both the LV and the RV base (dark blue and dark red regions). The simulation with the pericardium predicts the ventricular base will move the most, in agreement with the target end-systolic motion (light blue and light red regions).

2.2 The effect of the pericardium on systolic curvature, wall thickening and strain distribution

Figure 3 shows the percentage change in LV wall thickness (A) and change in curvature (B) of the LV endocardium from end-diastole to end-systole, projected on the LV endocardium. We calculated local curvature of the left ventricular endocardium using the method presented by Thomas and Chan (Thomas and Chan, 1989). This method fits a sphere to a patch of the endocardial surface. The curvature is then calculated as the

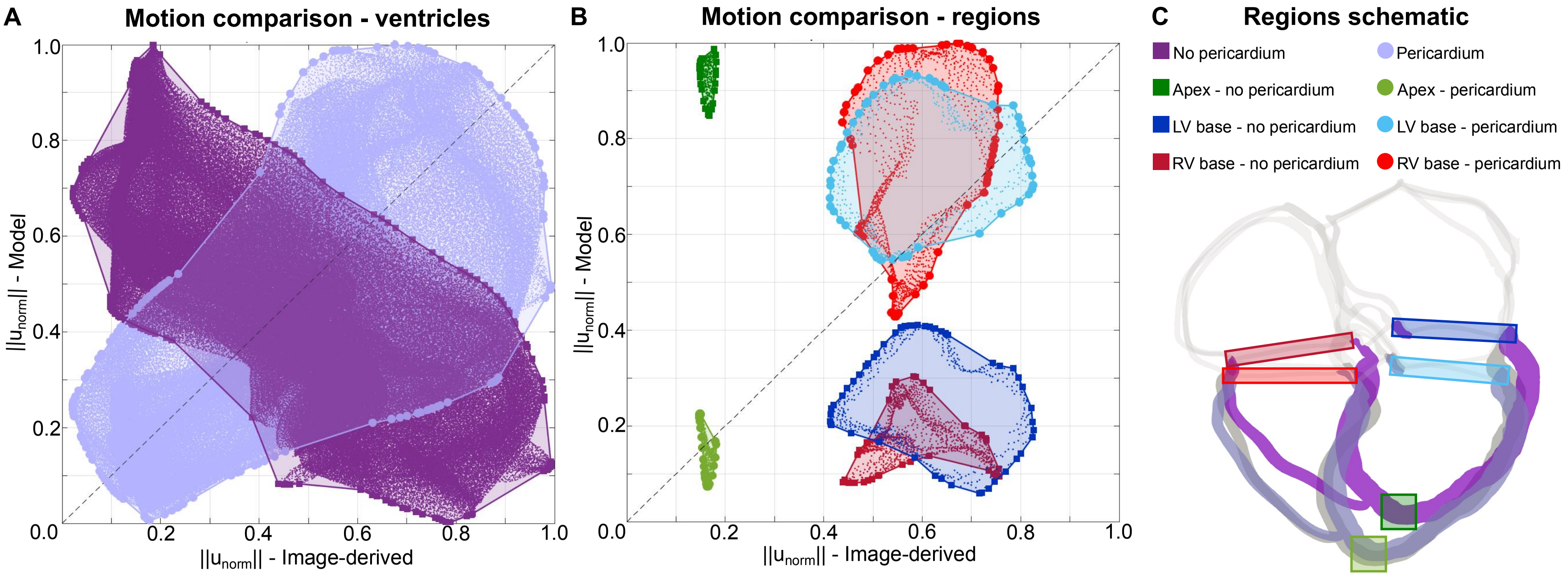

Figure 2: **Comparison of the end-systolic displacement - ventricles.** For each ventricular point, we computed the displacement from end-diastole for the end-systolic configuration estimated by the motion tracking, and the simulated end-systolic configuration simulated in the presence and in the absence of the pericardium. We then normalised all three datasets between 0 and 1. In sub-figure A, we plotted the normalised values for the simulations with (light-purple) and without (dark purple) the pericardium against the corresponding normalised value estimated by the motion tracking. We finally enclosed the point clouds with convex hulls. For visualisation purposes, we show the same data divided in different regions in sub-figure B: apex, left ventricle base and right ventricle base. Sub-figure C shows the legend of the different regions and the different colors for results in the presence and in the absence of the pericardium.

inverse of the radius of the fitted sphere. We chose a patch size of approximately 30mm in radius. The top and the bottom rows of both panels depict the quantities resulting from the simulations without (top row) and with (bottom row) the effect of the pericardium, respectively. We also show the distribution of the wall thickening and change in curvature on the triangles of the LV endocardium in the histograms on the right. The orange and the light-blue bars indicate the results for simulations without and with the pericardium, respectively. The pericardium causes a global decrease in wall thickening. This effect is more evident at the apex, where the estimated wall thickening is considerably lower with the effect of the pericardium (third and fourth panels). Large differences in end-systolic change in curvature were found between simulations with and without the effect of the pericardium. In the absence of the pericardium, the LV free wall and the septum show increased curvature as the chamber deforms to a more spherical shape, while at the apex the curvature change is negligible. On the other hand, in the presence of the pericardium curvature change in the free wall is decreased, as the change in shape of the epicardial surface is limited. At the septum the change in curvature is approximately zero, while it increases at the apex. Figure 6A and 6B show the values of wall thickening and change in curvature we obtained without the pericardium (x axis) plotted versus the values we obtained with the pericardium (y axis). The moderate correlation (R = 42.6%) of curvature changes in the presence and in the absence of the pericardium highlights the differences between the two simulations. We tested if the patch size for curvature estimation affected our results. Figure 4 shows the curvature change distribution in the presence (blue) and in the absence (orange) of the pericardium for the control patch radius (30mm), and for 20% patch bigger (right) or smaller (left) path radius. Our results show that the differences in curvature change distributions between simulation in the presence and in the absence of the pericardium are independent of the patch size for local curvature estimation.

To quantify the effect of the pericardium on end-systolic strain distribution the resulting end-systolic strain tensor was projected onto the local myofibre, sheet and normal-to-sheet orientation. Figure 5 shows results for the six components of the Cauchy-Green strain tensor in an apical, midwall and basal slice of the LV for the simulations with and without the effect of the pericardium. We also show the local distribution of the element-wise difference between the strain estimated without and with the effect of the pericardium, and the histograms of the distribution of local strains on the LV for the simulation without (orange) and with (light-blue) the effect of the pericardium. Both with and without the pericardium, the fibre strain is uniformly negative (Figure 6A) indicating uniform myofibre shortening, while shear strains E_{fs} and E_{fn} were small (Figure 6B and 6C). Our simulations show an inverse relationship between sheet and normal-to-sheet strains E_{ss} and E_{nn}. Regions with large and negative E_{ss} have large and positive E_{nn} and vice-versa. The pericardium changes strain distribution only at the apex (Figure 5D and 5F), where larger E_{ss} and E_{nn} were simulated in the absence of the pericardium. Figure 6C-H show the LV end-systolic strains projected in the fibre, sheet and normal-to-sheet directions obtained without the pericardium plotted versus the strains obtained with the pericardium. Our results show that there is a strong linear correlation for all quantities apart from the end-systolic curvature change. This indicates that the presence of the pericardium has a limited effect on strain distribution.

Our results indicate that the presence of the pericardium has limited effects on strain

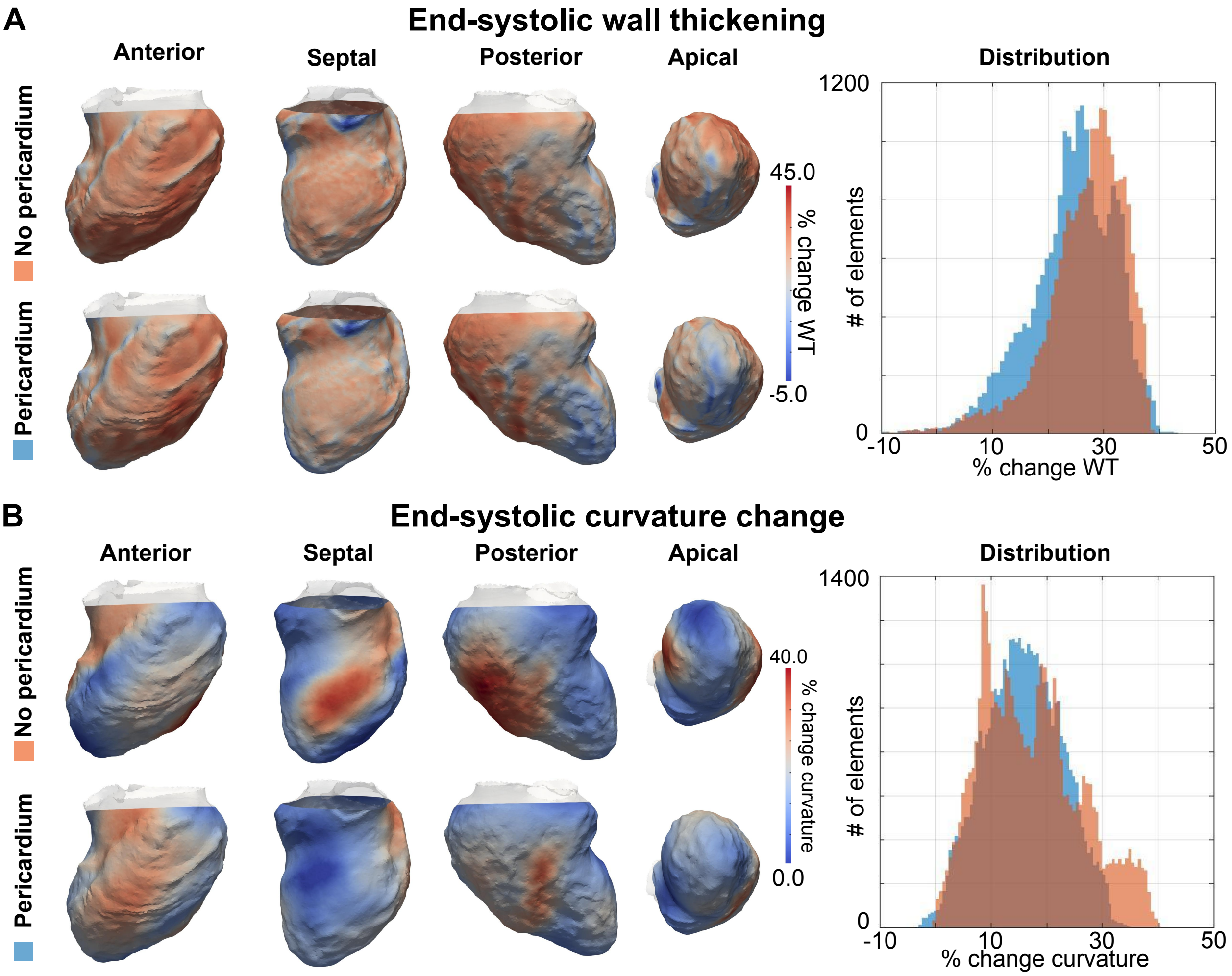

Figure 3: **A End-systolic wall thickening.** The percentage change in LV end-systolic wall thickness w.r.t. end-diastole simulated without (top row) and with (bottom row) was projected on the LV endocardial surface. We show an anterior, septal, posterior and apical view. On the right, the distribution over the triangles of the LV endocardium of the wall thickening is shown for the simulation without (orange) and with (light-blue) the effect of the pericardium. **B End-systolic change in curvature.** The percentage change in LV end-systolic curvature w.r.t. ED simulated without (top row) and with (bottom row) was projected on the LV endocardial surface. We show an anterior, septal, posterior and apical views. On the right, the distribution over the triangles of the LV endocardium of the change in curvature is shown for the simulation without (orange) and with (light-blue) the effect of the pericardium.

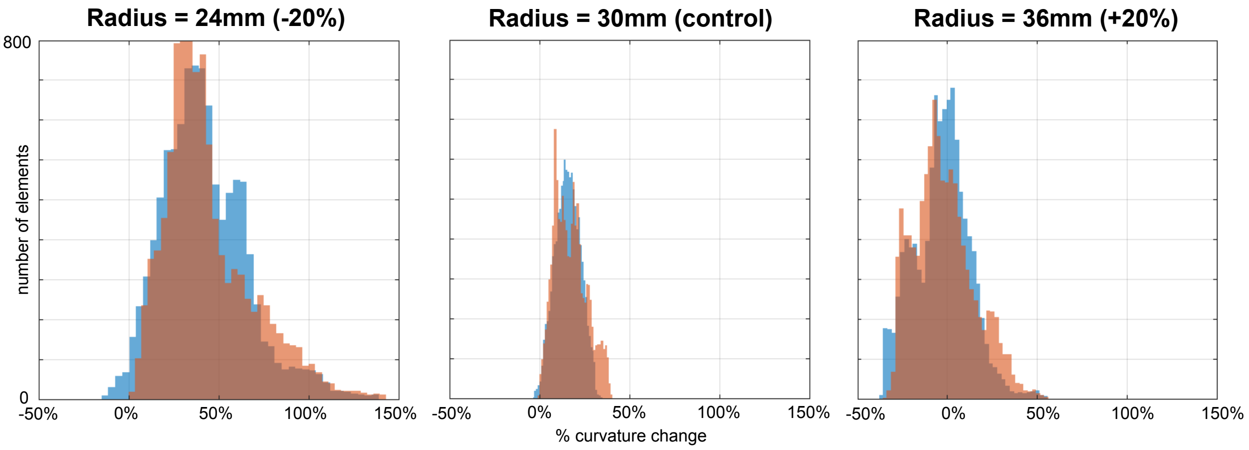

Figure 4: **Effect of patch size.** The histograms show percentage curvature change from end-diastole to end-systole in the presence (blue) and in the absence of the pericardium (orange). The figure in the center shows the distribution for the control patch radius (30mm). The figures on the left and on the right show the distribution of curvature change for patch radii of 24mm (-20%) and 36mm (+20%).

distribution, except at the apex. Both with and without the effect of the pericardium, our results for E_{ff}, E_{fs}, E_{fn} and E_{ss} agree with measurements reported in the literature (Costa et al., 1999). Small effects of different boundary conditions on strain distribution were also reported in the past (Peirlinck et al., 2019). Ponnaluri et al compared simulated average strains in a LV model in the presence and in the absence of the pericardium. They also reported limited differences in fibre and radial strains (Ponnaluri et al., 2019), consistent with our observations. Ponnaluri et al however observed significant differences in circumferential and longitudinal strains. These differences could be due to local myofibre orientation, as we used a rule based method and Ponnaluri et al used ex-vivo DTMRI measurements. Our results show that boundary conditions have a strong effect on displacement distribution, indicating that strain is a more robust measurement for model validation, particularly for simulations that do not include a pericardium boundary constraint.

Previous studies have described the role of orthotropic material laws in wall thickening (Costa et al., 1999). We studied the effect of ventricular myocardium orthotropy on end-systolic wall thickening, change in curvature and strain distribution. Figure 7 shows the comparison between results obtained with the Guccione law and with the Usyk law. In each panel, the x and y axes represent the same quantity resulting from the simulation with the transversely istropic law and with the orthotropic law, respectively. Therefore, if orthotropy had no effect on the results, the black points would lay on the red dashed line. The orange square and the light-blue diamond represent results for simulations without and with the effect of the pericardium, respectively. The green circle represents the element-wise difference between results obtained without and with the pericardium. We analysed: end-systolic percentage LV wall thickening without the pericardium (A), with the pericardium (B) and the difference between the two (C); end-systolic percentage curvature change without the pericardium (C), with the pericardium (D) and the difference

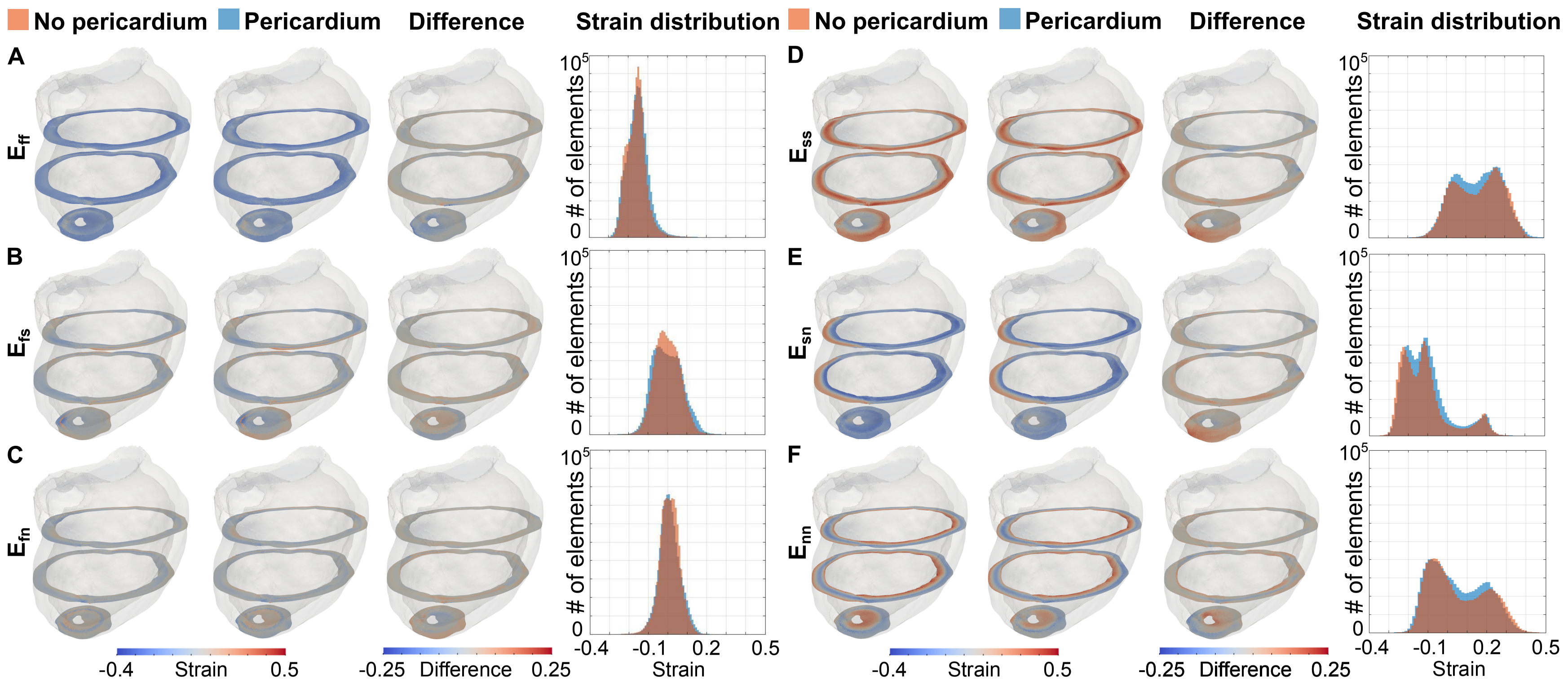

Figure 5: **Local strain distribution.** The images show the local distribution of the six components of the Cauchy-Green strain tensor on an apical, midwall and basal slice of the LV. We also show the local distribution of the difference between the simulation without and with the pericardium, and the histograms of the distribution of the strain components on the LV for the simulation without (orange) and with (light-blue) the pericardium.

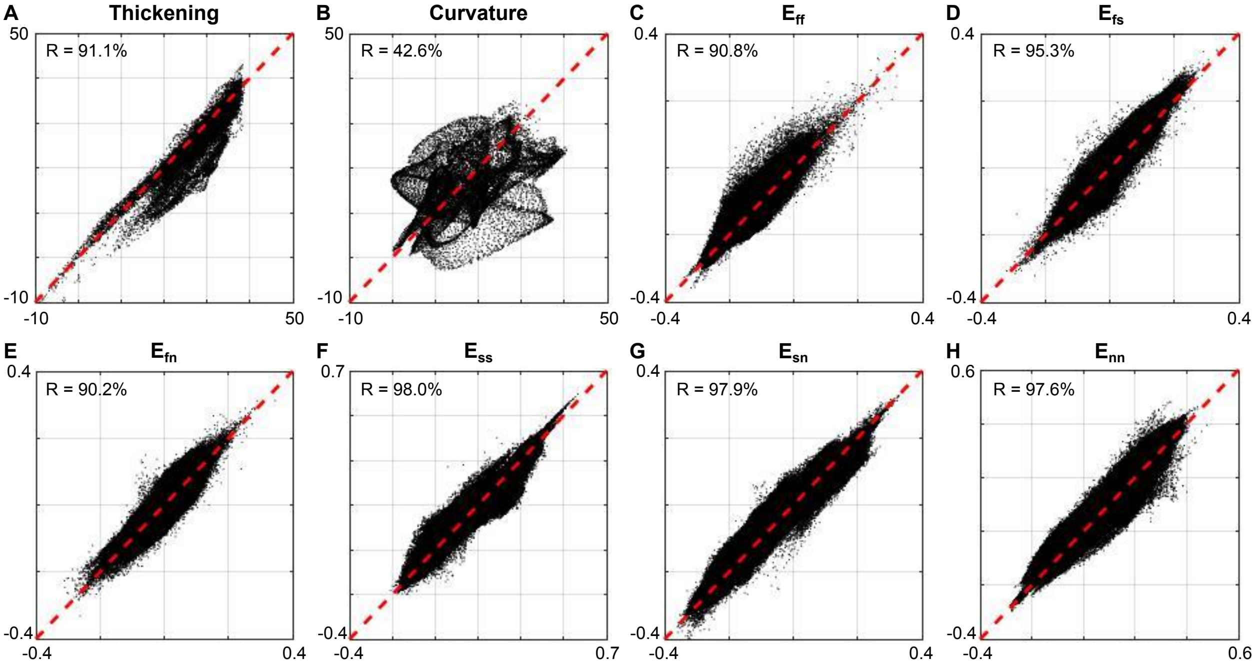

Figure 6: **Effect of the pericardium.** In all panels, the x and the y axes are the quantity of interest resulting from the simulation without and with the effect of the pericardium, respectively. Quantities we considered are: (**A**) LV percentage wall thickening at end-systole; (**B**) LV percentage curvature change at end-systole; (**C**-**H**) components of the Cauchy-Green strain tensor projected in the local fibre, sheet and normal-to-sheet directions.

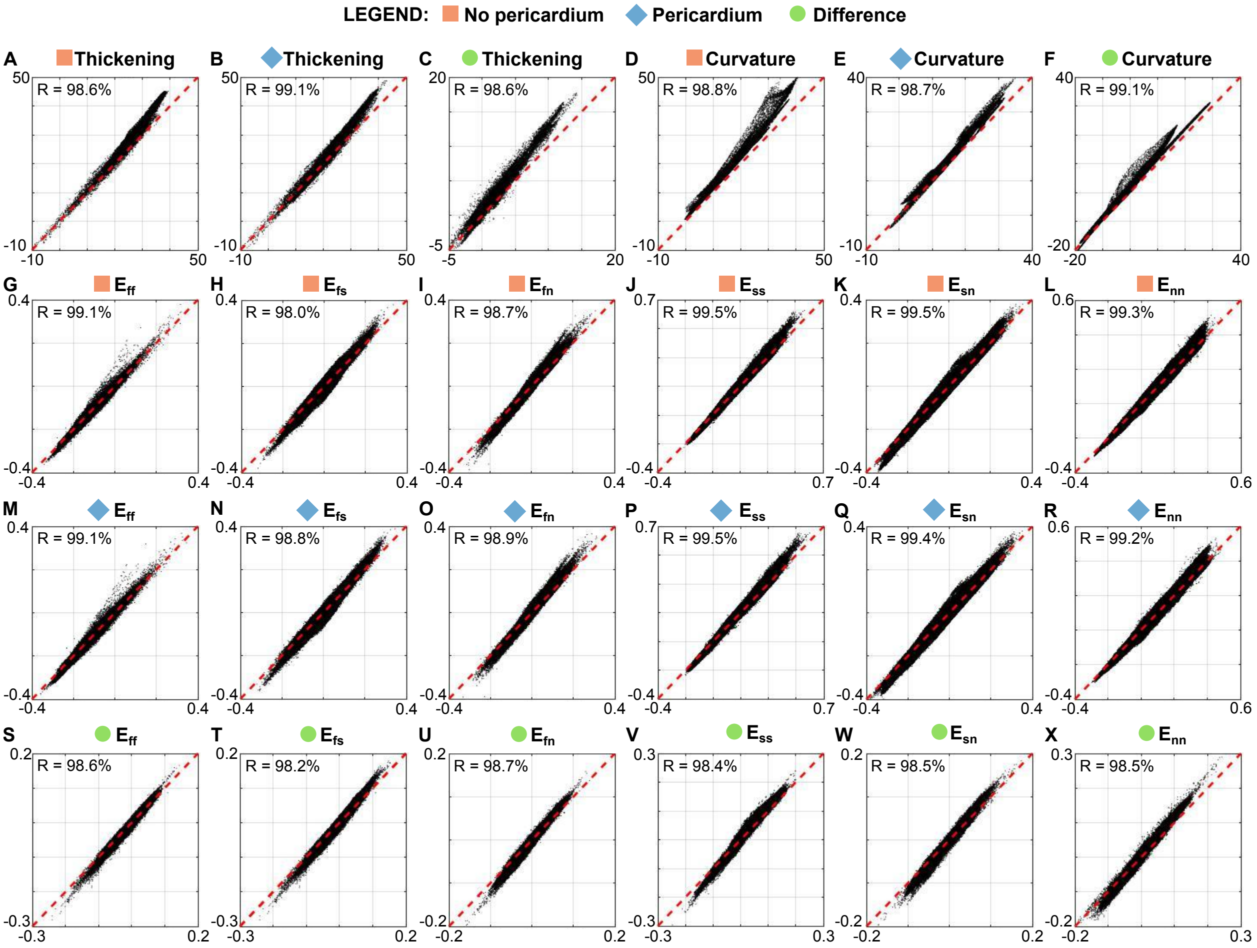

Figure 7: **Effect of orthotropy.** In all panels, the x and the y axes are the quantity of interest resulting from the simulation with a transversely isotropic material law and with an orthotropic material law, respectively. The orange square and the light-blue diamond circle represent results of simulations without and with the pericardium, respectively. The green circle instead represent the element-wise difference between the results without and with the effect of the pericardium. Quantities we considered are: (**A**-**C**) LV percentage wall thickening at end-systole; (**D**-**F**) LV percentage curvature change at end-systole; (**G**-**X**) components of the Cauchy-Green strain tensor projected in the local fibre, sheet and normal-to-sheet directions.

between the two (E); the six components of the Cauchy-Green strain tensor on the LV without the pericardium (G-L), with the pericardium (M-R) and the difference between the two (S-X). Our results show that using an orthotropic material law has a limited effect on end-systolic thickening, change in curvature and strain distribution.

References

Augustin, C.M., Crozier, A., Neic, A., Prassl, A.J., Karabelas, E., Ferreira da Silva, T., Fernandes, J.F., Campos, F., Kuehne, T., Plank, G., 2016. Patient-specific modeling of left ventricular electromechanics as a driver for haemodynamic analysis. EP Europace 18, iv121–iv129.

Behar, J.M., Rajani, R., Pourmorteza, A., Preston, R., Razeghi, O., Niederer, S., Adhya, S., Claridge, S., Jackson, T., Sieniewicz, B., et al., 2017. Comprehensive use of cardiac computed tomography to guide left ventricular lead placement in cardiac resynchronization therapy. Heart Rhythm 14, 1364–1372.

Beinart, R., Abbara, S., Blum, A., Ferencik, M., Heist, K., Ruskin, J., Mansour, M., 2011. Left atrial wall thickness variability measured by CT scans in patients undergoing pulmonary vein isolation. J Cardiovasc Electrophysiol 22, 1232–1236.

Blair, S.N., Goodyear, N.N., Gibbons, L.W., Cooper, K.H., 1984. Physical fitness and incidence of hypertension in healthy normotensive men and women. JAMA 252, 487–490.

Bols, J., Degroote, J., Trachet, B., Verhegghe, B., Segers, P., Vierendeels, J., 2013. A computational method to assess the in vivo stresses and unloaded configuration of patient-specific blood vessels. J Comput Appl Math 246, 10–17.

CIBC, 2016. Seg3d: Volumetric image segmentation and visualization. scientific computing and imaging institute (SCI), download from: http://www.seg3d.org. http://www.sci.utah.edu/cibc-software/seg3d.html .

Costa, K.D., Takayama, Y., McCulloch, A.D., Covell, J.W., 1999. Laminar fiber architecture and three-dimensional systolic mechanics in canine ventricular myocardium. American Journal of Physiology-Heart and Circulatory Physiology 276, H595–H607.

Crozier, A., Augustin, C.M., Neic, A., Prassl, A.J., Holler, M., Fastl, T.E., Hennemuth, A., Bredies, K., Kuehne, T., Bishop, M.J., Niederer, S.A., Plank, G., 2015. Image-based personalization of cardiac anatomy for coupled electromechanical modeling. Ann Biomed Eng 44, 58–70. URL: https://doi.org/10.1007%2Fs10439-015-1474-5, doi:10.1007/s10439-015-1474-5.

Di Martino, E.S., Bellini, C., Schwartzman, D.S., 2011. In vivo porcine left atrial wall stress: computational model. Journal of biomechanics 44, 2589–2594.

Flory, P., 1961. Thermodynamic relations for high elastic materials. Transactions of the Faraday Society 57, 829–838.

Fritz, T., Wieners, C., Seemann, G., Steen, H., Dössel, O., 2014. Simulation of the contraction of the ventricles in a human heart model including atria and pericardium. Biomech Model Mechanobiol 13, 627–641.

Guccione, J.M., McCulloch, A.D., Waldman, L.K., et al., 1991. Passive material properties of intact ventricular myocardium determined from a cylindrical model. J Biomech Eng 113, 42–55.

Gurev, V., Pathmanathan, P., Fattebert, J.L., Wen, H.F., Magerlein, J., Gray, R.A., Richards, D.F., Rice, J.J., 2015. A high-resolution computational model of the deforming human heart. Biomech Model Mechanobiol 14, 829–849.

Ho, S., Nihoyannopoulos, P., 2006. Anatomy, echocardiography, and normal right ventricular dimensions. Heart 92, i2–i13.

Hornỳ, L., Žitnỳ, R., Chlup, H., Macková, H., 2006. Identification of the material parameters of an aortic wall. Bull Appl Mech 2, 173–181.

Hyde, E.R., Behar, J.M., Claridge, S., Jackson, T., Lee, A.W.C., Remme, E.W., Sohal, M., Plank, G., Razavi, R., Rinaldi, C.A., et al., 2015. Beneficial effect on cardiac resynchronization from left ventricular endocardial pacing is mediated by early access to high conduction velocity tissue: An electrophysiological simulation study. Circ Arrhythm Electrophysiol , CIRCEP–115.

Hyde, E.R., Behar, J.M., Crozier, A., Claridge, S., Jackson, T., Sohal, M., Gill, J.S., O'NEILL, M.D., Razavi, R., Niederer, S.A., et al., 2016. Improvement of right ventricular hemodynamics with left ventricular endocardial pacing during cardiac resynchronization therapy. Pacing Clin Electrophysiol 39, 531–541.

Kerckhoffs, R.C.P., Bovendeerd, P.H.M., Prinzen, F.W., Smits, K., Arts, T., 2003. Intra and interventricular asynchrony of electromechanics in the ventricularly paced heart. J Eng Math 47, 201–216.

Knoll, F., Clason, C., Bredies, K., Uecker, M., Stollberger, R., 2012. Parallel imaging with nonlinear reconstruction using variational penalties. Magn Reson Med 67, 34–41.

Land, S., Niederer, S.A., 2018. Influence of atrial contraction dynamics on cardiac function. Int J Numer Method Biomed Eng 34, e2931.

Lee, A., Nguyen, U., Razeghi, O., Gould, J., Sidhu, B., Sieniewicz, B., Behar, J., Mafi-Rad, M., Plank, G., Prinzen, F., et al., 2019. A rule-based method for predicting the electrical activation of the heart with cardiac resynchronization therapy from non-invasive clinical data. Medical image analysis 57, 197–213.

Matsukubo, H., Matsuura, T., Endo, N., Asayama, J., Watanabe, T., 1977. Echocardiographic measurement of right ventricular wall thickness. a new application of subxiphoid echocardiography. Circulation 56, 278–284.

Mensel, B., Kühn, J.P., Schneider, T., Quadrat, A., Hegenscheid, K., 2013. Mean thoracic aortic wall thickness determination by cine MRI with steady-state free precession: validation with dark blood imaging. Acad Radiol 20, 1004–1008.

Nasopoulou, A., Shetty, A., Lee, J., Nordsletten, D., Rinaldi, C.A., Lamata, P., Niederer, S., 2017. Improved identifiability of myocardial material parameters by an energy-based cost function. Biomech Model Mechanobiol 16, 971–988.

Neic, A., Campos, F.O., Prassl, A.J., Niederer, S.A., Bishop, M.J., Vigmond, E.J., Plank, G., 2017. Efficient computation of electrograms and ECGs in human whole heart simulations using a reaction-eikonal model. J Comput Phys .

Niederer, S.A., Plank, G., Chinchapatnam, P., Ginks, M., Lamata, P., Rhode, K.S., Rinaldi, C.A., Razavi, R., Smith, N.P., 2010. Length-dependent tension in the failing heart and the efficacy of cardiac resynchronization therapy. Cardiovasc Res 89, 336–343.

Nordsletten, D.A., Niederer, S.A., Nash, M.P., Hunter, P.J., Smith, N.P., 2011. Coupling multi-physics models to cardiac mechanics. Prog Biophys Mol Biol 104, 77–88.

Ogden, R., 1978. Nearly isochoric elastic deformations: application to rubberlike solids. J Mech Phys Solids 26, 37–57.

Peirlinck, M., Sack, K.L., De Backer, P., Morais, P., Segers, P., Franz, T., De Beule, M., 2019. Kinematic boundary conditions substantially impact in silico ventricular function. International journal for numerical methods in biomedical engineering 35, e3151.

Ponnaluri, A.V., Verzhbinsky, I.A., Eldredge, J.D., Garfinkel, A., Ennis, D.B., Perotti, L.E., 2019. Model of left ventricular contraction: Validation criteria and boundary conditions, in: International Conference on Functional Imaging and Modeling of the Heart, Springer. pp. 294–303.

Schmid, H., O'Callaghan, P., Nash, M.P., Lin, W., LeGrice, I.J., Smaill, B.H., Young, A.A., J., H.P., 2008. Myocardial material parameter estimation: A non-homogeneous finite element study from simple shear tests. Biomechanics and Modeling in Mechanobiology 128, 742–750. doi:10.1115/1.2244576.

Strik, M., Regoli, F., Auricchio, A., Prinzen, F., 2012. Electrical and mechanical ventricular activation during left bundle branch block and resynchronization. J Cardiovasc Transl Res 5, 117–126.

Thomas, S.M., Chan, Y.T., 1989. A simple approach for the estimation of circular arc center and its radius. Computer Vision, Graphics, and Image Processing 45, 362–370.

Tian, L., Wang, Z., Liu, Y., Eickhoff, J.C., Eliceiri, K.W., Chesler, N.C., 2016. Validation of an arterial constitutive model accounting for collagen content and crosslinking. Acta Biomater 31, 276–287.

Usyk, T., Mazhari, R., McCulloch, A., 2000. Effect of laminar orthotropic myofiber architecture on regional stress and strain in the canine left ventricle. Journal of elasticity and the physical science of solids 61, 143–164.

Vigmond, E., Dos Santos, R.W., Prassl, A., Deo, M., Plank, G., 2008. Solvers for the cardiac bidomain equations. Prog Biophys Mol Biol 96, 3–18.

Vigmond, E.J., Hughes, M., Plank, G., Leon, L.J., 2003. Computational tools for modeling electrical activity in cardiac tissue. J Electrocardiol 36, 69–74.

Westerhof, N., Lankhaar, J.W., Westerhof, B.E., 2009. The arterial windkessel. Med Biol Eng Comput 47, 131–141.

Zapol, W.M., Snider, M.T., 1977. Pulmonary hypertension in severe acute respiratory failure. New Engl J Med 296, 476–480.

Zheng, Y., Barbu, A., Georgescu, B., Scheuering, M., Comaniciu, D., 2008. Four-chamber heart modeling and automatic segmentation for 3-d cardiac ct volumes using marginal space learning and steerable features. IEEE transactions on medical imaging 27, 1668–1681.
